# Distribution and Habitat Suitability of *Artemisia annua* in China Under Climate Change Scenarios

**DOI:** 10.1002/ece3.74236

**Published:** 2026-09-14

**Authors:** Fenguo Zhang, Dongqing Yan, Yanyue Mao, Zhusong Liu, Jiamin Peng, Guanghua Zhao, Yongji Wang

**Affiliations:** ^1^ School of Life Science, Shanxi Engineering Research Center of Microbial Application Technologies Shanxi Normal University Taiyuan Shanxi China; ^2^ School of Life Sciences South China Normal University Guangzhou China

**Keywords:** *Artemisia annua*, climate change, ENMeval, MaxEnt, niche modeling

## Abstract

*Artemisia annua* is a medicinal plant of high therapeutic and economic value, yet its wild resources have declined markedly in recent decades. Climate change is expected to further alter its geographical distribution, posing challenges to both conservation and sustainable utilization. Here, we applied a Maximum Entropy (MaxEnt) model that integrates climatic, topographic, and soil variables to assess the current and future suitable habitats of *A. annua* in China under three CMIP6 shared socioeconomic pathways (SSP126, SSP245, and SSP585) for the mid‐21st century (2050s) and late‐21st century (2090s). We hypothesized that winter temperature conditions and precipitation seasonality would be the principal climatic constraints on habitat suitability, and that future climate change would drive an overall contraction and northeastward shift of suitable areas, with stronger changes under higher‐emission scenarios. The model was optimized using the ENMeval framework by tuning the regularization multiplier and feature classes, thereby reducing overfitting and improving ecological realism. Model predictions indicate that the current suitable habitat of *A. annua* covers approximately 2.02 × 10^6^ km^2^, mainly distributed across central and southwestern China. Habitat suitability is primarily constrained by winter temperature conditions, precipitation seasonality, and elevation, highlighting the dominant role of climatic tolerance and topographic mediation in shaping its ecological niche. Under future climate scenarios, the total suitable area is projected to contract, with the most pronounced reductions occurring under the high‐emission SSP585 pathway. Spatially, suitable habitats are expected to shift northeastward, with the centroid migrating from northern Sichuan toward southern Shaanxi, reflecting a climate‐driven redistribution toward cooler regions. By distinguishing stable, *contracting*, and *expanding* zones of suitable habitat, this study provides a spatially explicit assessment of *A. annua* 's vulnerability and adaptive potential under climate change. Our findings offer ecological insights into the species' climate sensitivity and provide a scientific basis for dynamic conservation planning and sustainable industrial distribution under future environmental change.

## Graphical abstract

We used an optimized MaxEnt model to assess the current and future suitable habitats of *Artemisia annua* in China under CMIP6 climate scenarios. Suitable areas are projected to contract overall and shift northeastward, primarily constrained by winter temperature and elevation. These results highlight the species' climate sensitivity and provide a basis for adaptive conservation and sustainable utilization.

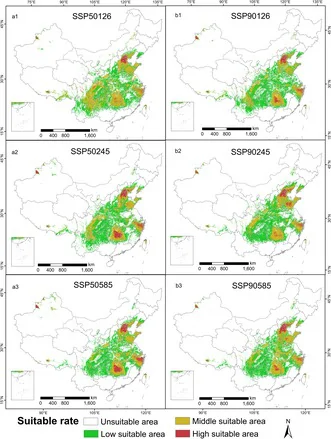

## 1 Introduction

Climate change has emerged as one of the most pervasive forces reshaping global biodiversity, profoundly influencing species distributions, community structure, and ecosystem functioning. Over the past century, global mean surface temperature has increased by approximately 1.09°C, with warming accelerating markedly in recent decades as a result of anthropogenic greenhouse gas emissions (Zhao et al. 2024). These rapid climatic shifts have already triggered widespread redistribution of plant species, commonly expressed as range contractions, poleward or elevational shifts, and increasing habitat fragmentation (Li et al. 2019; Pecl et al. 2017). Such changes pose growing challenges to ecosystem stability and biodiversity conservation, particularly for species with narrow climatic tolerances (Tian and Jiang 2015). Medicinal plants constitute a distinctive group within terrestrial ecosystems, combining ecological sensitivity with substantial socio‐economic importance. Many medicinal species occupy relatively narrow ecological niches and exhibit strong dependencies on specific climatic conditions, rendering them especially vulnerable to climate‐driven habitat alteration (Manish 2022). Climate‐induced redistribution of medicinal plants threatens not only the persistence and genetic diversity of wild populations but also the geographic authenticity (“*Daodi*”) and long‐term stability of medicinal resource supply (Rana et al. 2020; Shen et al. 2021). Consequently, clarifying how climate change reshapes the potential distribution of medicinal plants is critical for both ecological conservation and sustainable resource management.

*Artemisia annua* is a globally important medicinal plant and the primary natural source of artemisinin, a cornerstone compound in malaria treatment and an increasingly recognized agent with antiviral, anti‐inflammatory, and anticancer properties. Naturally distributed across temperate and subtropical regions, *A. annua* exhibits pronounced sensitivity to climatic conditions, particularly temperature and precipitation regimes (Long et al. 2015; Soni et al. 2022; Yuan et al. 2017). Specifically, its distribution is primarily constrained by low winter temperatures rather than high summer temperatures, indicating pronounced cold sensitivity that limits its establishment in colder temperate regions. In terms of moisture, the species favors environments with moderate precipitation seasonality, as excessive contrasts between dry and wet seasons impose physiological stress, whereas balanced seasonal regimes support stable growth and development. Previous studies have examined its distribution patterns at global and regional scales using a range of approaches, including geographic information systems, ecological niche models, and multi‐model ensemble frameworks (Ding et al. 2020). These efforts have provided valuable insights into current habitat suitability patterns and associated environmental drivers.

Despite this progress, several key knowledge gaps remain. First, most existing studies have focused on present or historical distribution patterns, whereas systematic projections under future climate scenarios remain limited, particularly at the national scale in China, one of the species' core distribution regions. Second, many species distribution modeling applications rely on default parameter settings, which may lead to model overfitting and inflated suitability estimates, thereby constraining ecological interpretability and predictive robustness. Third, relatively few studies have explicitly addressed the spatial reorganization of suitable habitats, including habitat stability, loss, and potential expansion zones—information that is essential for developing adaptive conservation and management strategies under climate change (Fan et al. 2009; Huang et al. 2010; Wang et al. 2022; Wang et al. 2024; Zhang, Wen, et al. 2018; Zhang et al. 2011).

Species distribution models (SDMs), and particularly the Maximum Entropy (MaxEnt) model, have become widely used tools for quantifying species–environment relationships and predicting potential distributions from presence‐background data (Phillips et al. 2006). When appropriately parameterized, MaxEnt can generate robust predictions even with limited occurrence records (Zhu et al. 2022). Recent advances in model optimization, such as the ENMeval framework, allow systematic tuning of regularization parameters and feature classes, thereby reducing model complexity and improving ecological realism. Coupling optimized SDMs with high‐resolution climate projections provides a powerful approach for exploring climate‐driven distribution dynamics and identifying areas of vulnerability and resilience (Chen et al. 2025; Fang et al. 2024).

In this study, we applied an optimized MaxEnt model incorporating climatic, topographic, and soil variables to assess the current and future suitable habitats of *A. annua* in China under three CMIP6 shared socioeconomic pathways (SSPs): SSP126 (a low‐emission sustainability scenario; 2.6 W/m^2^ by 2100), SSP245 (a medium‐emission intermediate scenario; 4.5 W/m^2^ by 2100), and SSP585 (a high‐emission, fossil‐fuel‐intensive development scenario; 8.5 W/m^2^ by 2100). These three SSPs encompass a broad range of potential climate trajectories, from strong mitigation to continued high emissions, and were applied for the mid‐century (2050s; 2041–2060) and late‐century (2090s; 2081–2100) periods. We hypothesized that: (1) optimization of MaxEnt parameters using ENMeval would reduce unnecessary model complexity and improve predictive robustness relative to the default configuration; (2) winter temperature conditions and precipitation seasonality would be the principal climatic constraints on the environmental suitability of *Artemisia annua* ; and (3) future climate change would lead to an overall contraction and northeastward shift of suitable areas, with stronger changes under higher‐emission scenarios. Specifically, we aimed to: (i) quantify the current spatial pattern of habitat suitability and identify dominant environmental drivers; (ii) project changes in habitat extent and spatial configuration under multiple future climate scenarios; and (iii) delineate stable, loss, and potential expansion zones to evaluate vulnerability and adaptive potential. By linking climate‐driven distribution shifts with ecological constraints, this study provides mechanistic insights into the climate sensitivity of *A. annua* and offers a scientific basis for dynamic conservation planning and sustainable resource utilization under ongoing environmental change.

## 2 Materials and Methods

### 2.1 Species Occurrence Data

Occurrence records of *Artemisia annua* were compiled from two publicly available biodiversity databases: the Chinese Virtual Herbarium (CVH; http://www.cvh.ac.cn) and the Global Biodiversity Information Facility (GBIF; https://www.gbif.org; accessed on January 24, 2024). For GBIF data retrieval, the search criteria were restricted to *Artemisia annua* L., with occurrence status set as “Present,” geographic region limited to China, records collected between 1900 and 2023, and only records containing geographic coordinates retained.

A total of 3496 occurrence records were initially obtained, including 1195 records from GBIF and 2301 records from CVH. All occurrence records were combined and subjected to a multi‐step quality‐control procedure before species distribution modeling. Records lacking geographic coordinates, containing obvious spatial errors, or with insufficient locality information were excluded. Coordinates obtained from online databases or georeferenced using Baidu Maps were converted from the BD‐09 coordinate system to the World Geodetic System 1984 (WGS84) to ensure consistency with environmental raster layers (Wang et al. 2025).

Data cleaning was performed using the CoordinateCleaner R package. This procedure was used to identify and remove records with low coordinate precision (e.g., coordinates rounded to whole degrees), duplicated localities, and potential georeferencing errors, including records located at country centroids, biodiversity institutions, or GBIF headquarters.

To reduce spatial sampling bias and spatial autocorrelation, spatial thinning was subsequently conducted using the spThin package (Aiello‐Lammens et al. 2015). Only one occurrence record within each 4.5‐km radius was retained, corresponding approximately to the spatial resolution of the environmental variables (2.5 arc‐min ≈4.5 km at the equator).

**FIGURE 1:**
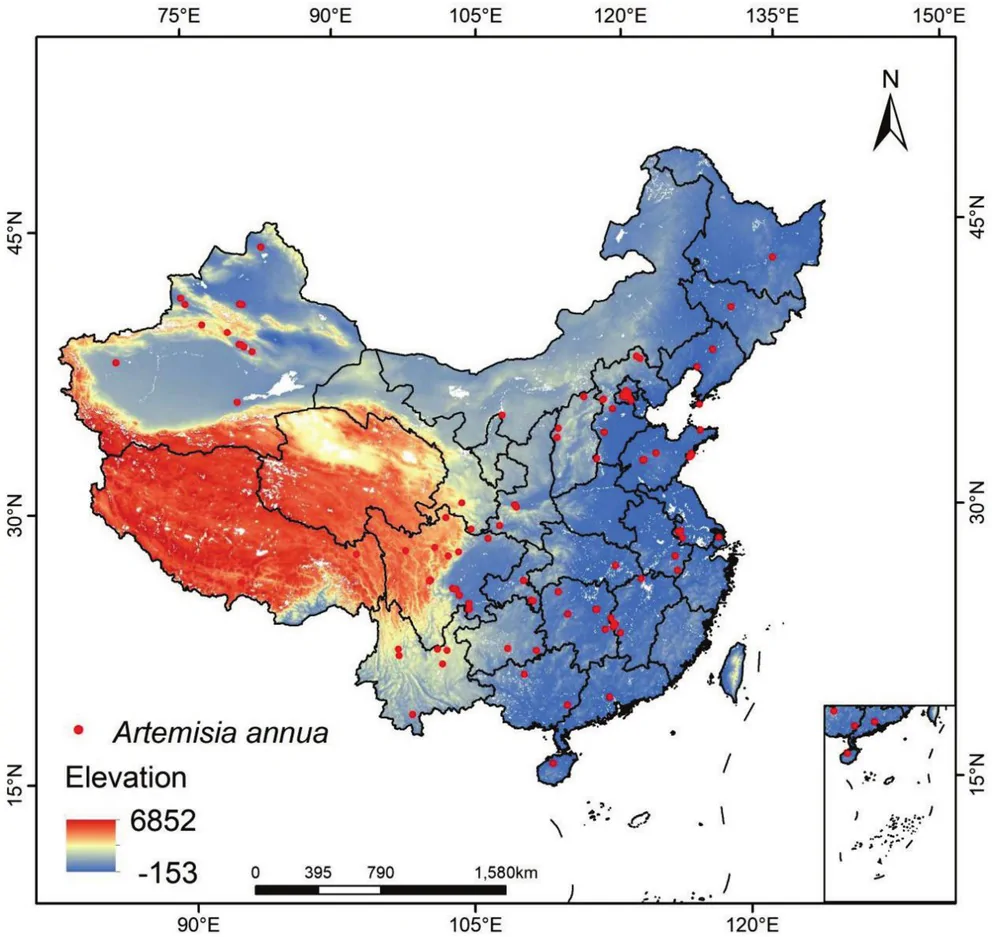
Geographical map of the distribution point of *Artemisia annua* within China. Each red dot represents a single, spatially independent occurrence record (e.g., an herbarium specimen or field observation) retained after quality filtering and spatial thinning at a 4.5‐km radius. The map delineates the species' documented range across central, eastern, and southwestern China, with notable clustering in the Qinling–Daba Mountains and the middle–lower Yangtze River basin.

After these quality‐control procedures and spatial filtering, 106 occurrence records were retained for model calibration and subsequent analyses (Figure 1). The final occurrence dataset and R scripts have been deposited in the Dryad Digital Repository (https://doi.org/10.5061/dryad.w6m905r4j).

### 2.2 Environmental Variables

A total of 37 environmental variables representing climatic, topographic, and soil conditions were initially considered. Bioclimatic variables for current conditions (1970–2000) and future climate scenarios (2050s: 2041–2060; 2090s: 2081–2100) were obtained from WorldClim version 2.1 at a spatial resolution of 2.5 arc‐min. Future climate projections were based on three CMIP6 shared socioeconomic pathways (SSP126, SSP245, and SSP585) (Zhang et al. 2024).

Topographic variables, including elevation, were derived from the Geospatial Data Cloud (http://www.gscloud.cn). Soil variables describing topsoil physicochemical properties were obtained from the Harmonized World Soil Database (HWSD). All environmental layers were resampled to the same spatial resolution and extent, and projected to the WGS84 coordinate system prior to analysis (Zhong et al. 2025).

To reduce multicollinearity among predictors and improve model interpretability, a two‐step variable selection procedure was applied. First, pairwise Pearson correlation coefficients were calculated, and for highly correlated variable pairs (|*r*| ≥ 0.7), only one ecologically representative variable was retained. Second, variance inflation factors (VIFs) were calculated using the **usdm** package, and variables with VIF values > 5 were excluded. Following this procedure, 12 environmental variables were retained for model construction (Table 1) (Muscarella et al. 2014; Naimi 2015; Zhang et al. 2017).

**TABLE 1:** The environmental aspects used in the modeling.

Type	Variable code	Environmental factor	Unit
Climatic factor	Bio1	Annual Mean Temperature	×10°C
	Bio5	Max Temperature of Warmest Month	×10°C
	Bio6	Min Temperature of Coldest Month	×10°C
	Bio11	Mean Temperature of Coldest Quarter	×10°C
	Bio15	Precipitation Seasonality	mm
	Bio16	Precipitation of Wettest Quarter	mm
Topographic factors	elev	Altitude	m
Soil factor	t_cec_soil	Cation exchange capacity of topsoil	%
	t_ece	Electrical conductivity of topsoil	dS/m
	t_grave	Gravel volume in topsoil	%
	t_oc	Organic carbon content in topsoil	%
	t_silt	Silt content in topsoil	%

### 2.3 Species Distribution Modeling

Habitat suitability of *A. annua* was modeled using MaxEnt version 3.4.4. Occurrence records were randomly split, with 75% used for model training and 25% reserved for model testing. Model outputs were generated using the cloglog transformation, which provides an estimate of habitat suitability interpreted as relative probability of presence (Phillips et al. 2017; Phillips and Dudík 2008).

Model performance was evaluated using the area under the receiver operating characteristic curve (AUC) and the 10% training omission rate. AUC values between 0.8 and 0.9 were considered indicative of good model performance (Wang et al. 2020).

### 2.4 Model Optimization

Under default settings (regularization multiplier RM = 1, feature classes FC = LQHPT), MaxEnt models tend to be overly complex, generating highly convoluted response curves that capture sampling noise and spatial bias rather than genuine ecological constraints. This overfitting inflates suitability estimates in heavily surveyed areas and compromises the model's ability to project distributions under future climatic conditions. To avoid these pitfalls and enhance ecological realism, we optimized model complexity using the ENMeval package in R (Muscarella et al. 2014). This framework allows simultaneous tuning of two parameters that jointly control model flexibility: the regularization multiplier (RM), which penalizes the inclusion of marginally informative predictors, and the feature classes (FC), which governs the shape of the fitted environmental response curves. We systematically varied RM from 0.5 to 4.0 (step 0.5) and evaluated six feature classes: L (linear), LQ (linear + quadratic), H (hinge), LQH (linear + quadratic + hinge), LQHP (linear + quadratic + hinge + product), and LQHPT (linear + quadratic + hinge + product + threshold), yielding 48 candidate models (Muscarella et al. 2014). Model complexity and performance were assessed using the corrected Akaike Information Criterion (AICc), average test AUC, and the 10% test omission rate (Ahmadi et al. 2023; Shabani et al. 2016).

The optimal model configuration was selected based on the minimum ΔAICc value (ΔAICc = 0), following established criteria that models with ΔAICc < 2 are considered statistically competitive (Phillips et al. 2017). The final optimized model was used for all subsequent projections under current and future climate scenarios.

### 2.5 Habitat Classification and Spatial Analysis

Continuous habitat suitability outputs were classified into four categories using a threshold‐based approach: unsuitable, low suitability, moderate suitability, and high suitability. The threshold distinguishing suitable from unsuitable habitat was defined using the 10th percentile training presence threshold, a conservative criterion commonly applied in presence‐only species distribution modeling (Liu et al. 2005).

Habitat suitability maps were generated in ArcGIS 10.8.1. Changes in suitable habitat extent under future climate scenarios were quantified by comparing raster outputs across time periods. Centroid positions of suitable habitats were calculated using the **SDMTools** package, and centroid shift distances were estimated using the **geosphere** package to quantify spatial redistribution trends (Liu et al. 2013).

To assess spatial dynamics of habitat change, current and future suitability maps were overlaid and reclassified into four categories: stable suitable areas, newly suitable areas, lost suitable areas, and persistently unsuitable areas. These spatial patterns were used to evaluate habitat stability, vulnerability, and potential expansion under climate change (Naimi and Araújo 2016; Zhang, Gao, and Qin 2018).

## 3 Results

### 3.1 Model Optimization and Performance

Using 106 occurrence records and 12 environmental variables, MaxEnt models with different parameter combinations were evaluated based on the corrected Akaike Information Criterion (AICc) and omission rates. The default parameter configuration (RM = 1.0, FC = LQHPT) exhibited relatively high model complexity (ΔAICc = 207.88) and a 10% training omission rate of 0.385.

**FIGURE 2:**
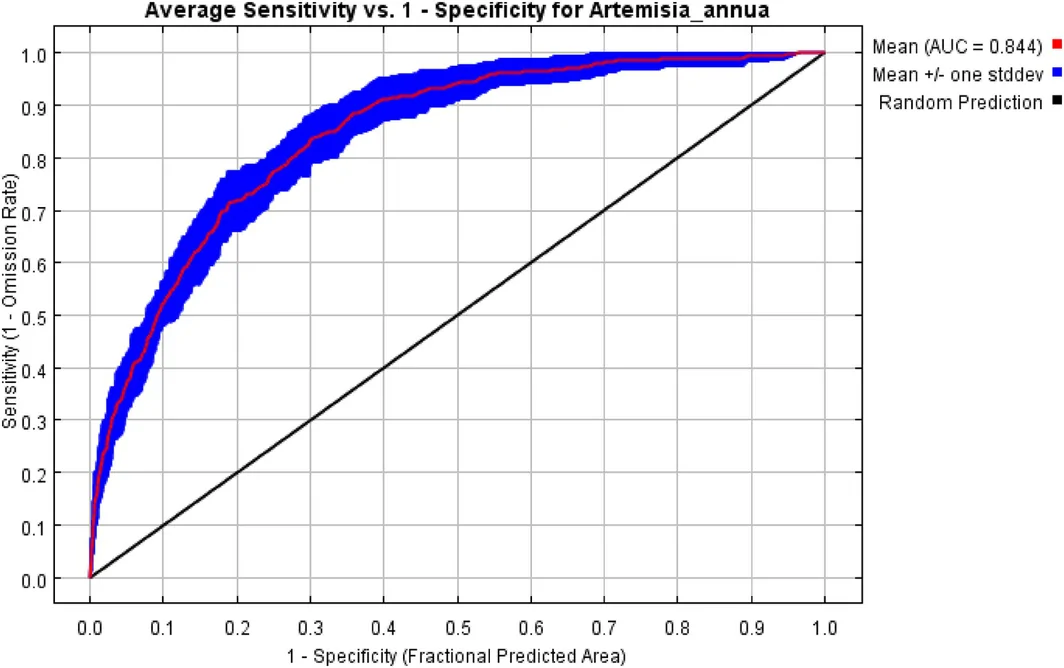
Receiver operating characteristic (ROC) response curve under the Maxent model. The x‐axis represents the false positive rate (1—specificity), and the y‐axis represents the true positive rate (sensitivity). The solid blue line shows the mean ROC curve averaged across ten cross‐validation replicates, with the surrounding light‐blue shaded area indicating ±1 standard deviation. The dashed red diagonal line corresponds to a random classifier (AUC = 0.5), where the model performs no better than chance. A curve that approaches the upper‐left corner reflects high sensitivity and low false positive rate, and thus higher overall model accuracy.

In contrast, the optimized model with RM = 1.5 and FC = H achieved the lowest AICc value (ΔAICc = 0) and substantially reduced the 10% training omission rate to 0.188 (Table 2). The mean training AUC of the optimized model was 0.844 (± 0.03), indicating good discriminatory ability (Figure 2). This parameter configuration was therefore selected for all subsequent habitat suitability analyses and future projections.

**TABLE 2:** Evaluation results of the Maxent model under different parameter settings.

Model evaluation	Feature classes	Regularization multiplier	Value of delta Akaike information criterion corrected	10% training omission rate
Default	LQHPT	1	207.88	0.385
Optimized	H	1.5	0	0.188

### 3.2 Current Habitat Suitability of *Artemisia annua* in China

**FIGURE 3:**
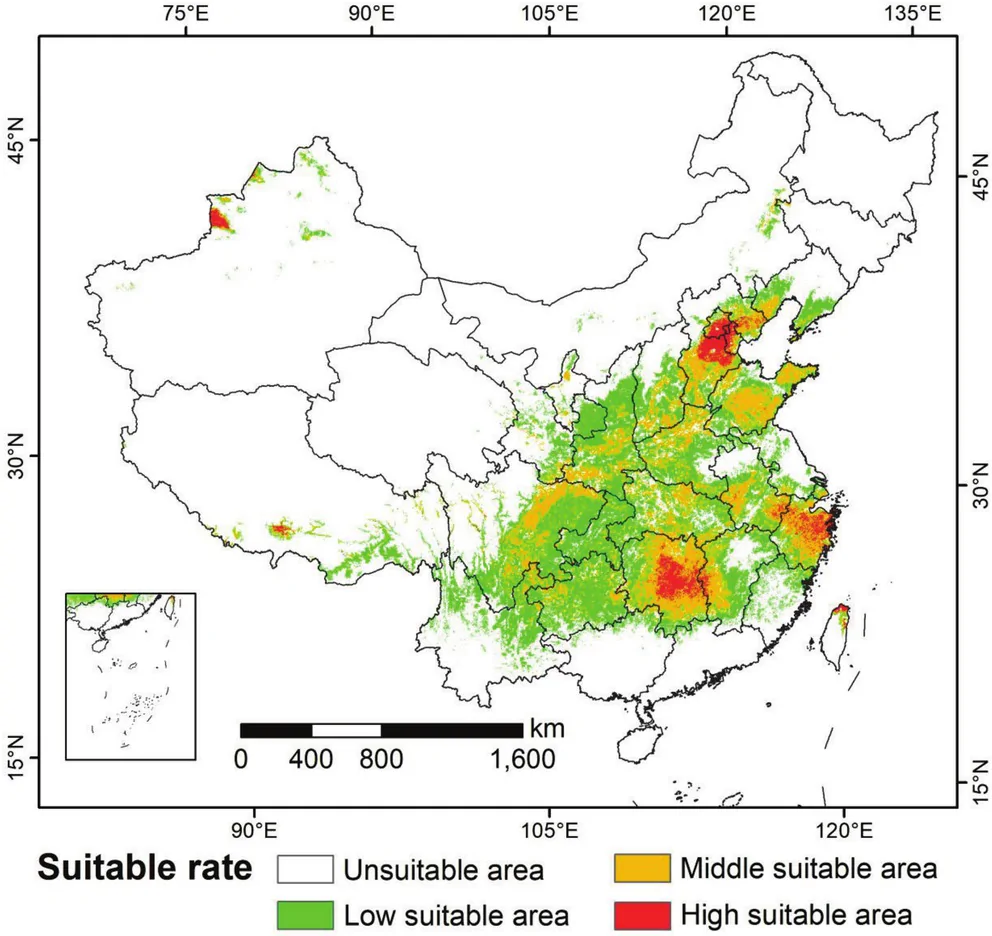
Potential environmental suitability of *Artemisia annua* in China via Maxent.

Under current climatic conditions (1970–2000), the predicted suitable habitat of *Artemisia annua* covered approximately 201.77 × 10^4^ km^2^ across China (Figure 3). Suitable areas were mainly distributed in central and eastern China, extending from the Qinling–Daba Mountains region toward the middle and lower reaches of the Yangtze River basin.

Within the total suitable area, low‐, moderate‐, and high‐suitability habitats accounted for 113.00 × 10^4^, 77.57 × 10^4^, and 11.20 × 10^4^ km^2^, respectively. Highly suitable habitats were spatially fragmented and occurred in discrete patches, whereas low‐ and moderately suitable habitats formed broader and more continuous distribution zones.

### 3.3 Projected Changes in Suitable Habitats Under Future Climate Scenarios

**FIGURE 4:**
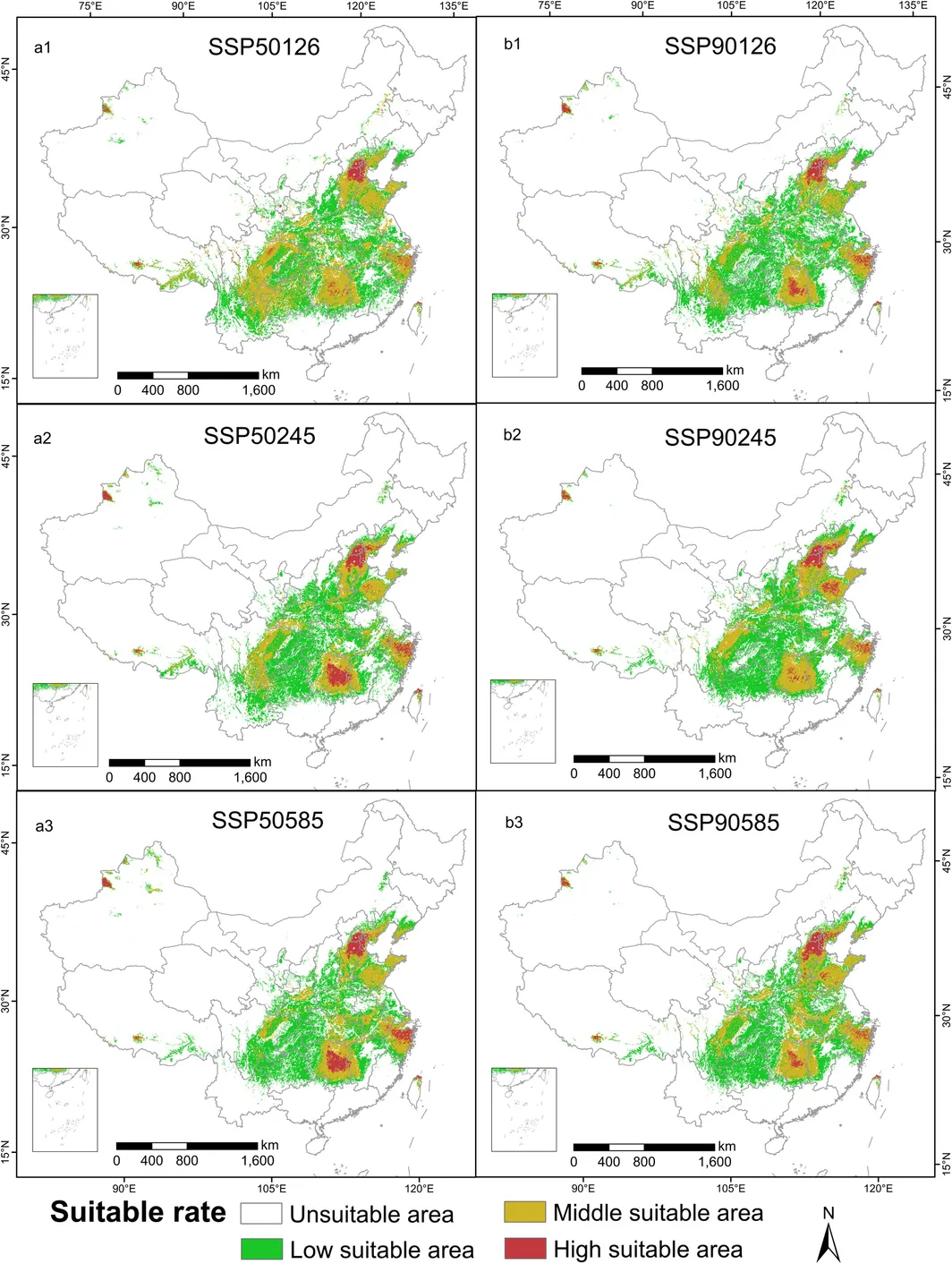
Suitable habitat distribution for *Artemisia annua* in China in the future under different climate change scenarios. Panels depict three SSPs (SSP126, SSP245, SSP585) for the 2050s (left column) and 2090s (right column). Suitability classes are high (red), moderate (yellow), low (green), and unsuitable (white).

Projections under future climate scenarios indicated an overall decline in the total suitable habitat area of *Artemisia annua* (Table 3; Figure 4). Relative to the current period, the total suitable area showed a slight increase under the SSP126 scenario in the 2050s (0.29%), but declined under all other scenarios, with reductions ranging from 9.56% to 16.95%.

**TABLE 3:** Suitable growing area of *Artemisia annua* in China, under different climates.

Climate change scenarios	Low‐grade suitable area	Moderately suitable area	Highly suitable area	Total suitable area
Current	113	77.57	11.2	201.77
SSP126‐2050s	108.19	86.61	7.55	202.36
SSP126‐2090s	114.57	55.56	8.95	179.07
SSP245‐2050s	111.11	59.13	12.69	182.93
SSP245‐2090s	104.25	59.17	9.44	172.86
SSP585‐2050s	102.9	54.15	12.61	169.66
SSP585‐2090s	99.08	61.61	9.59	170.28

*Note:* Scenarios in the future ×10^4^ km^2^.

The most pronounced habitat contraction occurred under SSP585, with the total suitable area declining to 169.66 × 10^4^ km^2^ by the 2050s before rebounding slightly to 170.28 × 10^4^ km^2^ by the 2090s. Although this increase was marginal (0.62 × 10^4^ km^2^, +0.37%), it contrasted with the continuing declines projected under SSP126 and SSP245 over the same period. Moderate‐suitability habitats exhibited the largest absolute reductions across scenarios, whereas changes in highly suitable habitats were smaller and showed greater inter‐scenario variability.

Spatially, suitable habitats consistently shifted toward northeastern China under all future climate scenarios (Figure 4). Habitat losses were most evident along the southern margins of the current distribution range, while newly suitable areas emerged mainly along the northern and northeastern edges.

### 3.4 Centroid Shifts of Suitable Habitats

**FIGURE 5:**
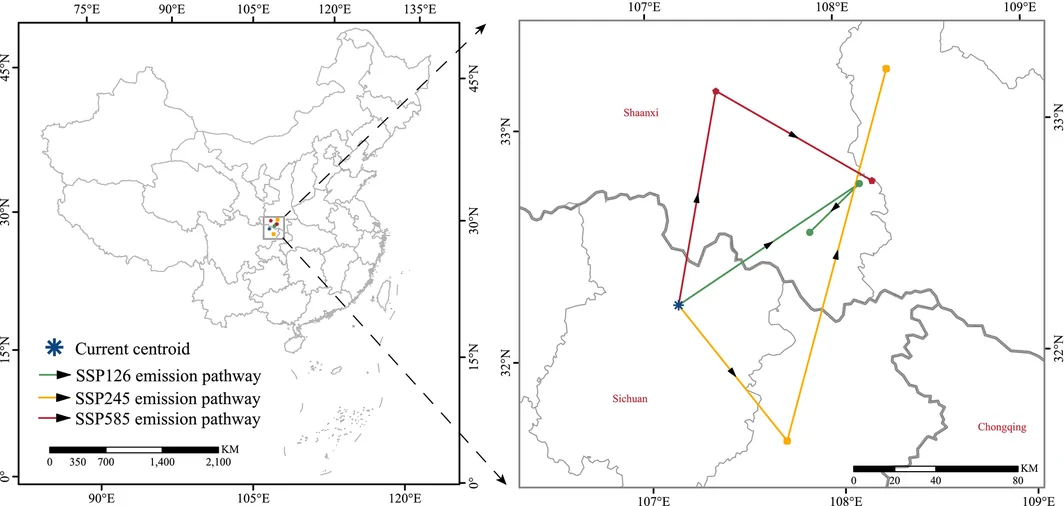
Geographical distribution changes of the centroid of the suitable growing area of *Artemisia annua* under different climate change scenarios.

The centroid of the current suitable habitat for *Artemisia annua* was located in northern Sichuan Province (107.15° E, 32.24° N; Figure 5). Under future climate scenarios, centroid positions shifted predominantly northeastward, although both the direction and magnitude of shifts varied among scenarios.

Under the SSP585 scenario, the centroid migrated approximately **192.86 km** by the 2090s, moving from northern Sichuan toward southern Shaanxi. The SSP245 scenario produced the largest centroid displacement, reaching **269.99 km** by the 2090s. In contrast, centroid shifts under the SSP126 scenario were smaller, with a maximum displacement of **140.66 km**.

### 3.5 Environmental Drivers of Habitat Suitability

Among the 12 environmental variables, four predictors contributed more than 10% to the MaxEnt model: mean temperature of the coldest quarter (bio11, 28.7%), precipitation seasonality (bio15, 17.2%), precipitation of the wettest quarter (bio16, 14.3%), and annual mean temperature (bio1, 13.0%) (Table 4). Together, these variables accounted for 73.4% of the total contribution.

**TABLE 4:** Contribution rate and importance of environmental variables.

Environmental variable	Contribution (%)	Importance (%)
Annual mean temperature	13	15.6
Max temperature of warmest month	0.1	2.4
Min temperature of coldest month	6.2	3.3
Mean temperature of coldest quarter	28.7	17.4
Precipitation seasonality	17.2	13.2
Precipitation of wettest quarter	14.3	6
Altitude	8.3	23.8
Cation exchange capacity of topsoil	0.5	0.7
Electrical conductivity of topsoil	2	3.5
Gravel volume in topsoil	1.1	0.9
Organic carbon content in topsoil	5.1	7
Silt content in topsoil	3.3	6.3

Permutation importance analysis further identified elevation (23.8%), bio11 (17.4%), bio1 (15.6%), and bio15 (13.2%) as the most influential predictors, indicating that both climatic and topographic factors strongly shaped habitat suitability.

**FIGURE 6:**
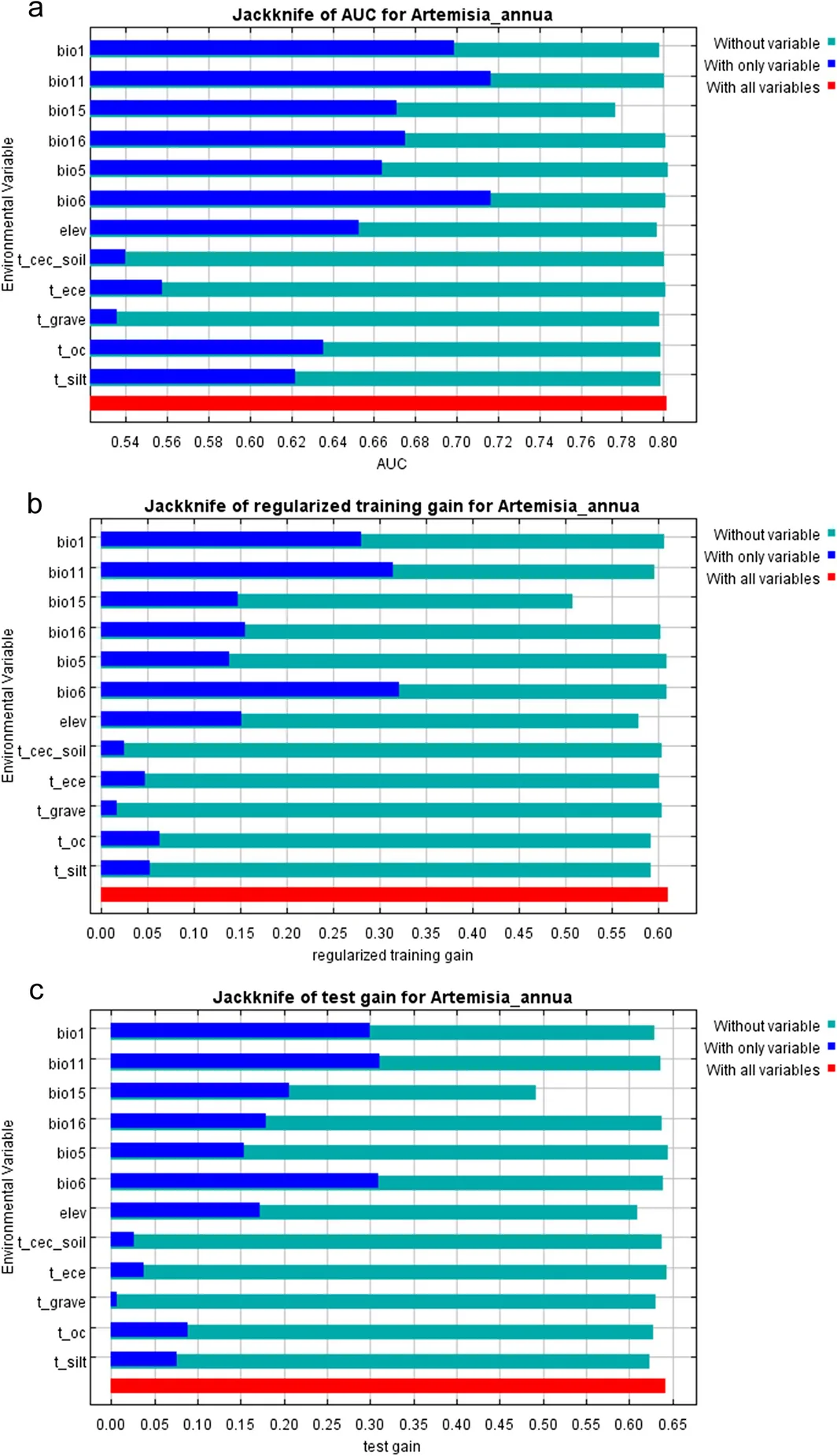
Results of jackknife evaluations of the predictor variables, using AUC on the test data (a), regularized training (b), and testing gain (c) in the MaxEnt model for *Artemisia annua*.

**FIGURE 7:**
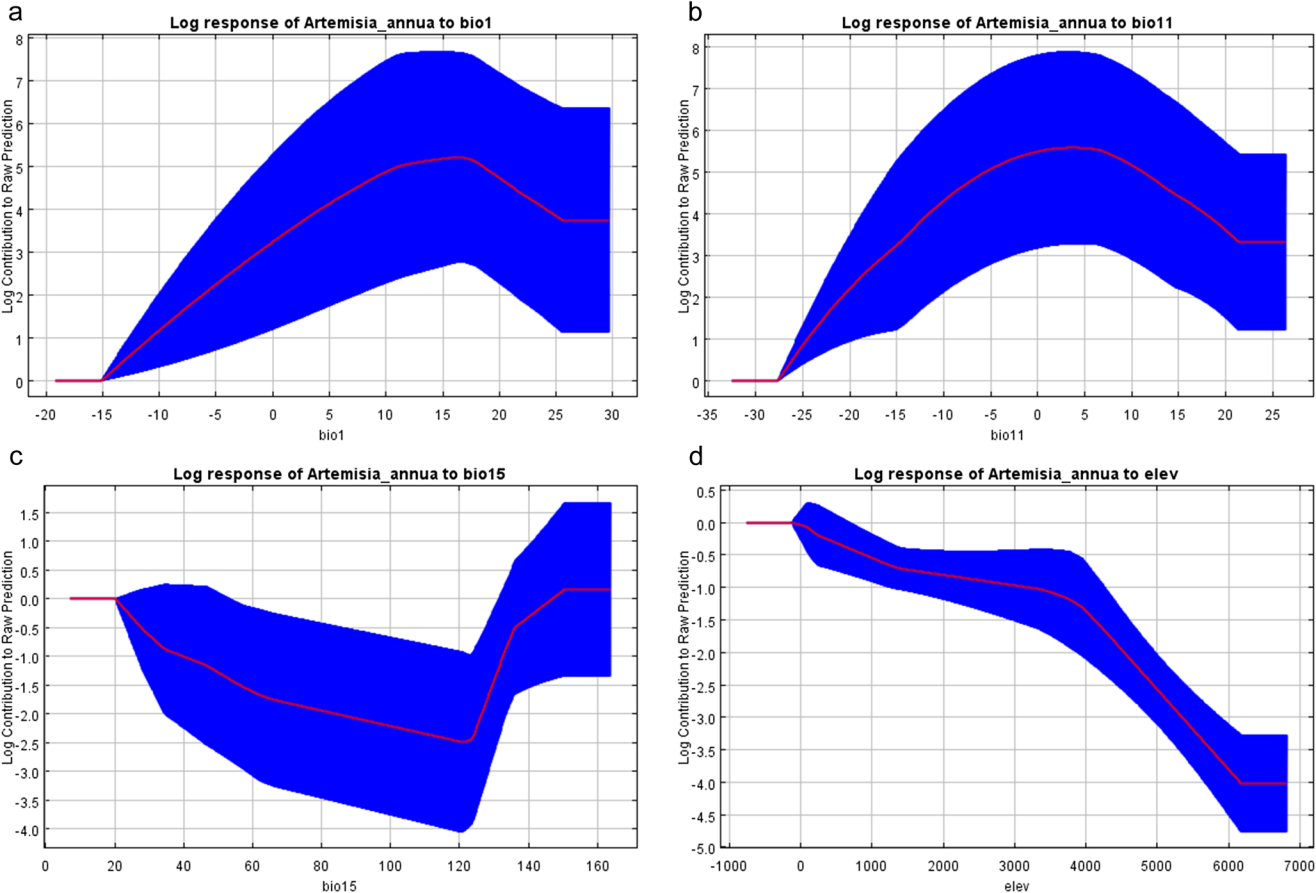
Single factor response curve of the current climate. Response curves showing the relationships between key environmental predictors and the habitat suitability of *Artemisia annua* under current climatic conditions. Each panel represents a single predictor, with the solid blue line indicating the mean predicted suitability across 10 replicate runs, and the shaded area representing ±1 standard deviation. The x‐axis shows the value of the environmental variable, and the y‐axis shows the predicted probability of occurrence.

Jackknife tests showed that models trained using bio11, bio1, bio15, or elevation alone achieved relatively high gains and AUC values, whereas excluding soil variables resulted in only minor reductions in model performance (Figure 6). Response curves revealed that habitat suitability increased with warmer winter temperatures and moderate precipitation seasonality, and followed a unimodal pattern along the elevational gradient, with optimal suitability occurring between 1000 and 3000 m (Figure 7).

### 3.6 Spatial Dynamics of Habitat Stability, Loss, and Gain

**FIGURE 8:**
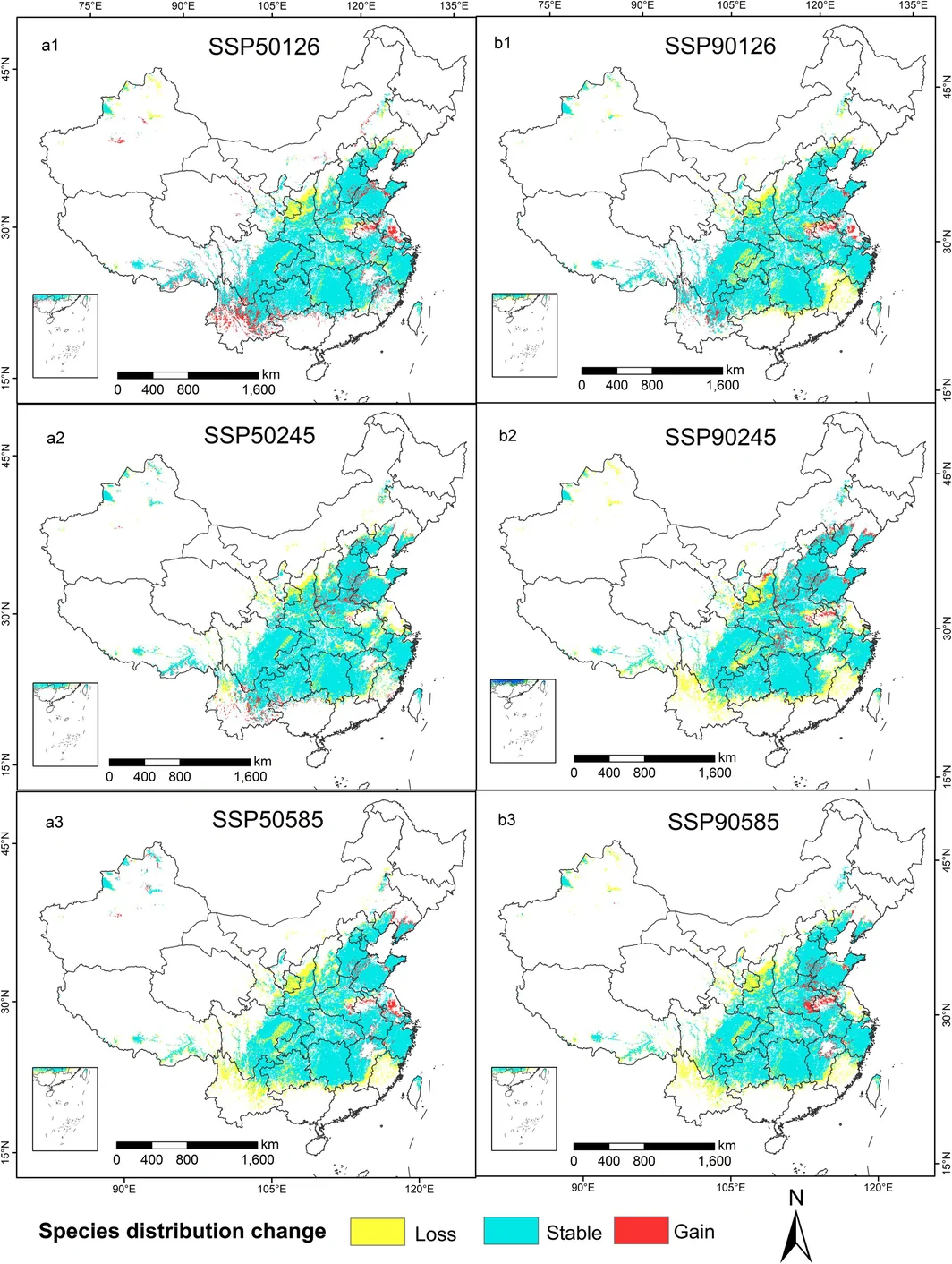
Spatial changes in *Artemisia annua* in China in the future under different climate change scenarios. Panels correspond to SSP126, SSP245, and SSP585 for the 2050s (left column) and 2090s (right column). Within each panel, areas are classified into four categories: Stable suitable (blue, suitable in both current and future conditions), loss (yellow, currently suitable but projected to become unsuitable), and gain (red, newly suitable in the future).

Overlay analyses of current and future habitat suitability maps revealed pronounced spatial reorganization of *Artemisia annua* habitats under climate change (Figure 8). Stable suitable areas accounted for 83.06%–93.84% of the current suitable habitat across scenarios, whereas habitat loss ranged from 10.61% to 21.39% (Table 5).

**TABLE 5:** Changes in the distribution area of *Artemisia annua* in different periods under climate change scenarios.

Period	Climate scenario	Habitat area (×10^4^ km^2^)	Loss (×10^4^ km^2^)	Stable (×10^4^ km^2^)	Gain (×10^4^ km^2^)	Species range change (%)	Percentage loss (%)	Percentage gain (%)
Current		201.77						
2041–2060	SSP126	202.36	21.40	189.34	23.41	0.99	10.61	11.6
	SSP245	182.93	30.87	179.87	11.59	−9.56	15.3	5.74
	SSP585	169.66	42.26	168.30	8.08	−16.95	20.95	4
2081–2100	SSP126	179.07	34.03	176.71	10.68	−11.58	16.87	5.29
	SSP245	172.86	43.15	167.59	12.07	−15.41	21.39	5.98
	SSP585	170.28	42.23	168.51	9.30	−16.32	20.93	4.61

Newly suitable habitats represented 4.00%–11.60% of the total suitable area and were primarily located near the northern and central portions of the current distribution range. In contrast, habitat losses were concentrated along the southern margins, particularly under high‐emission scenarios.

## 4 Discussion

### 4.1 Ecological Implications of Model Optimization

Optimizing model complexity is a critical step in species distribution modeling, particularly when projecting species responses under future climate scenarios where extrapolation into novel climatic spaces is inherently high. By applying the ENMeval framework, this study substantially reduced model overfitting while maintaining robust predictive performance, as evidenced by lower omission rates and smoother response curves (Ye et al. 2020; Zhao et al. 2021). Overfitted models typically produce jagged, multi‐modal response curves that lack biological realism, whereas our optimized model generated smoother, monotonic or unimodal curves that better reflect known physiological thresholds. These improvements enhanced ecological interpretability by allowing the model to capture fundamental environmental constraints rather than fitting localized sampling artifacts (Li et al. 2018; Ouyang et al. 2022; Phillips et al. 2017).

Notably, the hinge‐only model (FC = H) substantially outperformed the default configuration (FC = LQHPT). Hinge features act as piecewise linear splines, capturing threshold‐like responses without imposing a fixed parametric shape. This flexibility is ecologically meaningful: it accommodates the abrupt suitability declines observed in *A. annua* below critical winter temperatures or beyond optimal precipitation seasonality (Figure 7). That a simpler hinge‐only model achieved markedly lower ΔAICc (0 vs. 207.88) and omission rate (0.188 vs. 0.385) confirms that the default settings overfit by fitting complex feature types to noise rather than to genuine niche constraints. This finding reinforces the need to tailor model complexity to the species and dataset, and demonstrates that optimized, parsimonious models can enhance both predictive accuracy and ecological interpretability.

The close correspondence between predicted suitable habitats and the known distribution range of *Artemisia annua* further supports the reliability of the optimized model. This finding underscores that appropriate parameter tuning is not merely a technical refinement, but a prerequisite for deriving ecologically meaningful inferences from species distribution models, especially in the context of climate change assessments (Fan et al. 2009; Huang et al. 2010; Wang et al. 2024; Zhang, Wen, et al. 2018; Zhang et al. 2011).

### 4.2 Climatic and Topographic Constraints Shaping the Distribution of *Artemisia annua*

Our results identify winter temperature as the primary climatic constraint governing the distribution of *Artemisia annua* , with the mean temperature of the coldest quarter exerting the strongest influence on habitat suitability. This pattern reflects the species' sensitivity to cold stress during seed dormancy and early spring emergence, which limits its establishment in colder temperate regions. The sharp decline in suitability under low winter temperatures highlights a clear physiological boundary shaping the species' geographical range (Ferreira and Janick 1996). This dominance of winter temperature over summer temperature is ecologically meaningful: as an annual plant, *A. annua* completes its life cycle within a single growing season, making survival during the critical overwintering‐to‐emergence transition far more consequential for population persistence than tolerance of summer heat extremes. Comparable patterns have been documented for other temperature‐sensitive medicinal species in China—for instance, *Gentiana rigescens* exhibits distributional constraints governed more strongly by winter thermal minima than by growing‐season temperatures, reflecting a shared physiological filter among temperate medicinal plants (Shen et al. 2021). The minimal contribution of maximum summer temperature (bio5, 0.1%) further supports this interpretation, suggesting that high summer temperatures rarely exceed the species' upper thermal tolerance within its current range, whereas low winter temperatures frequently fall below critical survival thresholds.

Elevation also emerged as a key determinant of habitat suitability, despite its lower contribution relative to climatic variables. Acting as an integrative factor, elevation indirectly modulates local temperature regimes, moisture availability, and radiation conditions. The unimodal elevational response observed in this study—with optimal suitability occurring between 1000 and 3000 m—suggests that *A. annua* occupies a relatively narrow vertical niche, consistent with its frequent occurrence in hilly and montane landscapes. This mid‐elevation preference has been similarly observed in other montane medicinal plants, such as *Panax notoginseng*, whose distribution is concentrated in a narrow elevational band where thermal and moisture conditions jointly optimize growth and secondary metabolite accumulation (Zhan et al. 2022). For *A. annua* , the elevational constraint likely reflects a balance between temperature‐dependent germination success at lower altitudes and cold‐stress limitation at higher altitudes. Together, winter temperature and elevation define a core ecological envelope within which *A. annua* can persist.

Precipitation seasonality and wet‐season precipitation further influenced habitat suitability, indicating that *A. annua* favors environments with moderate seasonal variability in water availability. Excessive contrasts between dry and wet seasons may impose physiological stress, whereas balanced precipitation regimes support stable growth during critical developmental periods (Long et al. 2015; Soni et al. 2022). This preference for moderate seasonality is consistent with the species' native distribution in monsoonal China, where predictable wet–dry cycles have historically synchronized germination timing and vegetative growth with water availability. As climate change amplifies precipitation extremes—lengthening dry spells and intensifying wet‐season rainfall—the resulting decoupling of phenological cues from moisture availability may pose an underappreciated threat to population stability.

### 4.3 Climate‐Driven Redistribution and Range Dynamics

Projections under future climate scenarios reveal that suitable habitats of *Artemisia annua* are likely to undergo an overall contraction accompanied by a consistent northeastward shift. Such redistribution patterns align with widely documented global trends of poleward and elevational range shifts driven by climate warming (Chen et al. 2011). Notably, both habitat loss and centroid displacement intensified under higher emission scenarios, emphasizing the species' sensitivity to the magnitude and rate of climatic change (Phillips et al. 2017).

The slight rebound in total suitable area observed under SSP585—from 169.66 × 10^4^ km^2^ in the 2050s to 170.28 × 10^4^ km^2^ in the 2090s—does not represent an overall range expansion, but rather reflects the non‐linear and spatially contrasting nature of climate forcing under the highest emission pathway. Rapid, sustained warming progressively degrades habitat suitability along the low‐latitude, low‐elevation southern margins, while simultaneously creating newly climatically suitable areas at higher latitudes and elevations. As the centroid of suitable habitat shifts markedly northeastward, these gains at the northern edge partially offset the extensive southern losses. However, this offset should be interpreted with caution: the newly suitable areas are generally smaller and more fragmented than the core habitats being lost, and MaxEnt projections do not explicitly model dispersal constraints and may therefore overestimate the species' capacity to colonize newly suitable areas. The small magnitude of change (0.62 × 10^4^ km^2^) further suggests that the net habitat contraction may stabilize or reverse non‐monotonically over time, rather than proceeding linearly.

The observed spatial reorganization reflects a dynamic trade‐off between habitat loss along the southern margins of the current distribution and potential gains at cooler northern edges. However, newly suitable areas were generally smaller and more fragmented than existing core habitats. This fragmentation may constrain population connectivity and limit the species' ability to fully track shifting climatic conditions, even where climatic suitability increases (Fang et al. 2024).

### 4.4 Habitat Stability, Vulnerability, and Adaptive Potential

By distinguishing stable, loss, and expansion zones, this study provides a spatially explicit framework for assessing the vulnerability and adaptive potential of *Artemisia annua* under climate change. Stable suitable areas, predominantly located in the Qinling–Daba Mountains region, may represent potential refugial areas warranting conservation attention. These regions are therefore likely to play a critical role in conserving genetic diversity and maintaining population resilience. The ecological significance of stable refugia extends beyond their demographic function: populations persisting in these areas may retain higher genetic diversity and local adaptations that are essential for species' long‐term evolutionary potential under environmental change. For medicinal plants specifically, refugial populations often harbor unique chemotypes with distinct pharmaceutical properties, making their conservation doubly important for both biodiversity and medicinal resource sustainability (Shen et al. 2021). Moreover, the Qinling–Daba Mountains have been identified as a significant ecological corridor facilitating plant dispersal and species exchange between the Tibetan Plateau and the East China plains (Jiang et al. 2025). This corridor function further enhances the conservation value of stable suitable areas by promoting gene flow and population connectivity across the landscape, thereby strengthening the long‐term resilience of *A. annua* populations in the face of ongoing climate change.

In contrast, areas identified as losing suitability—mainly along the southern margins of the current distribution—represent climate‐sensitive zones where populations may face heightened risks of decline or local extirpation. Expansion zones at the northern and northeastern edges of the distribution indicate potential future habitats, although successful colonization will depend on dispersal capacity, landscape connectivity, and interactions with resident biota. Habitat loss along the southern margins was associated with projected changes in winter temperature and precipitation seasonality. As the mean temperature of the coldest quarter (bio11) exceeds the species' optimal thermal range (Figure 7; Table 4), the cold‐stress boundary that once constrained *A. annua* 's poleward distribution shifts northward, rendering low‐latitude areas thermally unsuitable during seed dormancy, germination, and early seedling establishment. Concurrently, projected increases in precipitation seasonality (bio15) over southern China amplify wet–dry contrasts, imposing moisture stress that further degrades habitat suitability in these regions. Conversely, newly suitable areas emerge at the northern and northeastern edges mainly because winter warming releases the low‐temperature limitation that historically excluded the species from cooler regions. As bio11 and bio1 rise into the suitable thermal windows identified in the response curves, and precipitation seasonality remains comparatively moderate, these areas become progressively more favorable, although they tend to be smaller, more fragmented, and disconnected from the current core distribution (Figures 4 and 8).

### 4.5 Implications for Conservation and Sustainable Utilization

The contrasting outcomes among emission pathways highlight the fundamental role of climate mitigation in shaping future habitat availability for *Artemisia annua* . While low‐emission scenarios may moderate habitat contraction and allow more gradual redistribution, high‐emission trajectories are likely to impose rapid habitat losses that exceed the species' adaptive capacity.

From an ecological management perspective, these findings support the adoption of a dynamic conservation framework. Priority should be given to protecting stable suitable areas as long‐term refuges, implementing monitoring and early‐warning strategies in vulnerable loss zones, and cautiously evaluating expansion zones for future cultivation or assisted migration. Similar climate‐adaptive approaches have been proposed for threatened plant species, where species distribution models and projected range losses were integrated to identify climate refugia, conservation priorities, and candidate sites for assisted colonization (Casazza et al. 2021). Such adaptive approaches acknowledge that species distributions are inherently dynamic and must be managed in concert with ongoing environmental change.

### 4.6 Limitations and Future Research Directions

Several limitations should be acknowledged. This study assumes static soil and topographic conditions and does not explicitly incorporate dispersal constraints, biotic interactions, or land‐use change, all of which may influence realized distributions. *Artemisia annua* produces numerous small achenes that are likely dispersed mainly over short distances by gravity and wind, whereas occasional long‐distance transport may also occur through animals or human activities. Consequently, the colonization of fragmented and geographically isolated newly suitable areas may be limited by dispersal constraints. In addition, uncertainties inherent in climate projections and model assumptions may affect the magnitude of predicted distributional shifts.

It should also be noted that some occurrence records did not coincide exactly with areas classified as suitable in the current prediction, whereas some highly suitable areas lacked documented occurrence records. Such mismatches do not necessarily indicate poor model performance. First, the environmental predictors were analyzed at a spatial resolution of 2.5 arc‐min; therefore, grid‐cell averages may not fully represent the fine‐scale microhabitat conditions at individual collection localities. Second, the 10th percentile training presence threshold is relatively conservative and may classify some marginal occurrence sites as unsuitable. Third, predicted suitability represents potential environmental suitability rather than actual occupancy. Areas predicted to be highly suitable but lacking occurrence records may remain unoccupied because of limited dispersal, incomplete sampling, land‐use constraints, biotic interactions, or historical processes. Accordingly, targeted field surveys should be conducted in both apparently unsuitable occurrence sites and highly suitable but unrecorded areas to validate and refine future predictions.

*Artemisia annua* is primarily wind‐pollinated, and its tiny seeds (achenes; ~0.55–0.8 mm; thousand‐grain weight ~0.035 g) are predominantly wind‐dispersed. Their small size, relatively smooth surface, and lack of a well‐developed pappus suggest that most seeds deposit within short to intermediate distances from the parent plant, although a small fraction may travel farther under favorable wind conditions. This predominantly short‐range dispersal likely constrains the species' capacity to track shifting climatic niches, underscoring the need to incorporate dispersal limitations into future modeling efforts.

Like most correlative species distribution models, MaxEnt projections commonly assume relative niche conservatism over time. Plants possess an intrinsic ability to adapt to changing environments through physiological adjustments and evolutionary processes. Our projections represent potential distribution shifts under the assumption that the species' current climatic niche remains static. However, if *A. annua* populations harbor adaptive genetic variation, they might acclimate or adapt to future conditions, potentially altering the realized distribution. Therefore, our results should be interpreted as a conservative estimate of risk under climate change. Future research integrating common garden experiments or genome‐environment association analyses is critical to elucidate the species' adaptive capacity and refine predictions.

Moreover, agricultural expansion, urbanization, and habitat degradation may lead to habitat loss or fragmentation that either outweighs or interacts with climatic effects. In subsequent research, we will incorporate dynamic land‐use scenarios to further enhance predictive accuracy. Beyond land‐use change, future research should also integrate dispersal processes, and population‐level physiological responses would further refine predictions. Combining species distribution models with genetic, demographic, or experimental approaches may provide deeper insights into the adaptive capacity of *Artemisia annua* under accelerating climate change.

## 5 Conclusions

By optimizing model complexity and integrating climatic, topographic, and soil variables, this study provides a robust assessment of the current and future habitat suitability of *Artemisia annua* in China. Our results demonstrate that winter temperature conditions, particularly the mean temperature of the coldest quarter, together with elevation, constitute the primary ecological constraints shaping the species' geographical distribution.

Projections under CMIP6 climate scenarios indicate that future climate change will likely lead to an overall contraction of suitable habitats for *A. annua* , accompanied by a pronounced northeastward shift in distribution. While high‐emission pathways exacerbate habitat loss and spatial reorganization, lower‐emission scenarios mitigate but do not eliminate these trends. Such redistribution patterns suggest increasing fragmentation of suitable habitats and heightened vulnerability of populations located along the southern margins of the current geographical range.

By explicitly delineating stable, loss, and potential expansion zones, this study highlights spatial heterogeneity in vulnerability and adaptive potential under climate change. Stable suitable areas may serve as long‐term refuges for sustaining population persistence and genetic diversity, whereas loss zones warrant early‐warning and adaptive management strategies. Expansion zones, although climatically suitable in the future, require cautious evaluation due to uncertainties associated with dispersal limitations and ecological interactions.

Overall, our findings underscore the necessity of transitioning from static to dynamic conservation and management approaches for *Artemisia annua* . Integrating climate‐informed distribution projections into conservation planning can support the sustainable utilization of this important medicinal species while enhancing its resilience to ongoing environmental change. These results represent projected changes in environmental suitability rather than direct evidence of future occupancy or population persistence.

## Author Contributions

**Fenguo Zhang:** conceptualization (equal), writing – original draft (equal). **Dongqing Yan:** methodology (equal), writing – review and editing (equal). **Yanyue Mao:** data curation (equal), formal analysis (equal), writing – review and editing (equal). **Zhusong Liu:** data curation (equal), methodology (equal), resources (equal). **Jiamin Peng:** software (equal), validation (equal). **Guanghua Zhao:** writing – review and editing (equal). **Yongji Wang:** funding acquisition (equal), supervision (equal).

## Funding

This work was supported by the project for Local Science and Technology Development Guided by Central Government Guides Local Science and Technology Development Fund (YDZJSX2025D060), General Teaching Reform and Innovation Project of Higher Education Institutions in Shanxi Province (J20230615), and Research Project Supported by Shanxi Scholarship Council of China (2023‐110, 2024‐089).

## Conflicts of Interest

The authors declare no conflicts of interest.

## Data Availability

The data that support the findings of this study are available in the Dryad Digital Repository at https://doi.org/10.5061/dryad.w6m905r4j.
